# 10271 How The Kidney Reacts to Nutritional Changes?

**DOI:** 10.1017/cts.2021.751

**Published:** 2021-03-30

**Authors:** Dana Bielopolski, Andrea Ronning, Jonathan N. Tobin, Rhonda G. Kost

**Affiliations:** 1 Rockefeller University; 2 Clinical Directors Network

## Abstract

ABSTRACT IMPACT: Understanding the mechanism underlying the DASH diet may shed light on the physiologic process by which nutrition influences blood pressure and potentially lead the way to new therapeutics that target ion channels. OBJECTIVES/GOALS: Hypertension is a disease of the westernized world, as it stems from lifestyle habits. Lower salt consumption reduces blood pressure, yet DASH diet is much more effective, lowering blood pressure as efficiently as one anti-hypertensive drug. The precise mechanism through which DASH achieves its effect is not understood, and this is the project goal. METHODS/STUDY POPULATION: We hypothesize that exposing hypertensive volunteers to a high potassium and low sodium DASH diet will change the composition of renal ion channel in an aldosterone-dependent manner, leading to excretion of both sodium and potassium and a reduction in blood pressure. To assess how the nutritional change changes ion channel composition in the kidneys’ epithelium in aldosterone-induced manner, we will monitor urine exosomes, which contain epithelial cell membranes. We designed an in-hospital nutritional studies recruiting 20 volunteers. Patients will consume carefully designed menu, and measurements will be collected daily: blood pressure, biologic samples including blood and urine electrolytes, aldosterone, and urine for exosomes. RESULTS/ANTICIPATED RESULTS: We have collected data from 5 research volunteers so far.following exposure to the high potassium diet, Aldosterone blood levels increased while blood level of both potassium and sodium was maintained within normal limits. Urinary ratio of electrolytes, sodium:potassium was reversed 5-7 days following nutritional change from 6 to 1. Both manual and automated 24-hour blood pressure measurements confirmed blood pressure reduction following nutritional change. The following illustrates the impact the diet had on a participant’s 24-hour ambulatory blood pressure. Daily mean blood pressure was reduced from 120/76 mmHG to 112/68, mean awake blood pressure was reduced from 125/80 mmHG to 117/72 mmHG, and mean sleep blood pressure was reduced from 112/69 to 103/60 mmHG DISCUSSION/SIGNIFICANCE OF FINDINGS: Understanding the mechanism underlying the DASH diet may shed light on the physiologic process by which nutrition influences blood pressure and potentially lead the way to new therapeutics that target ion channels. By introducing our participants to a healthier lifestyle, they could maintain lower blood pressure without requiring medication.

